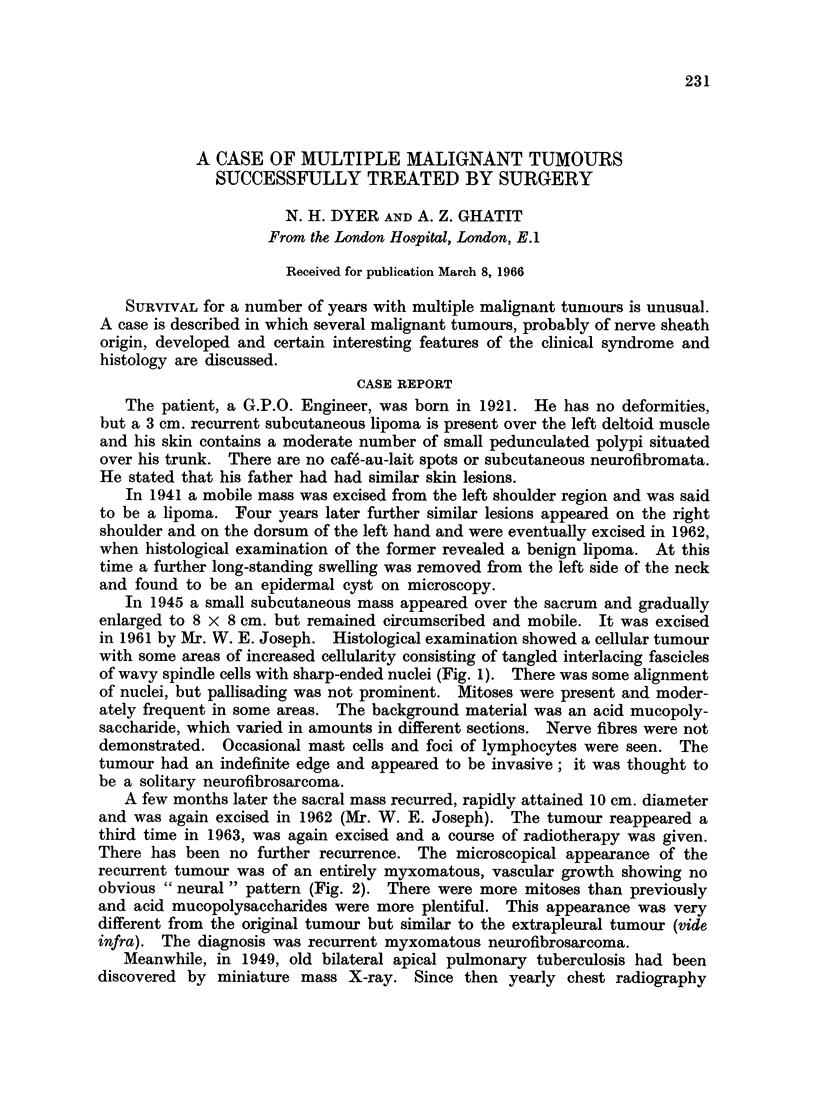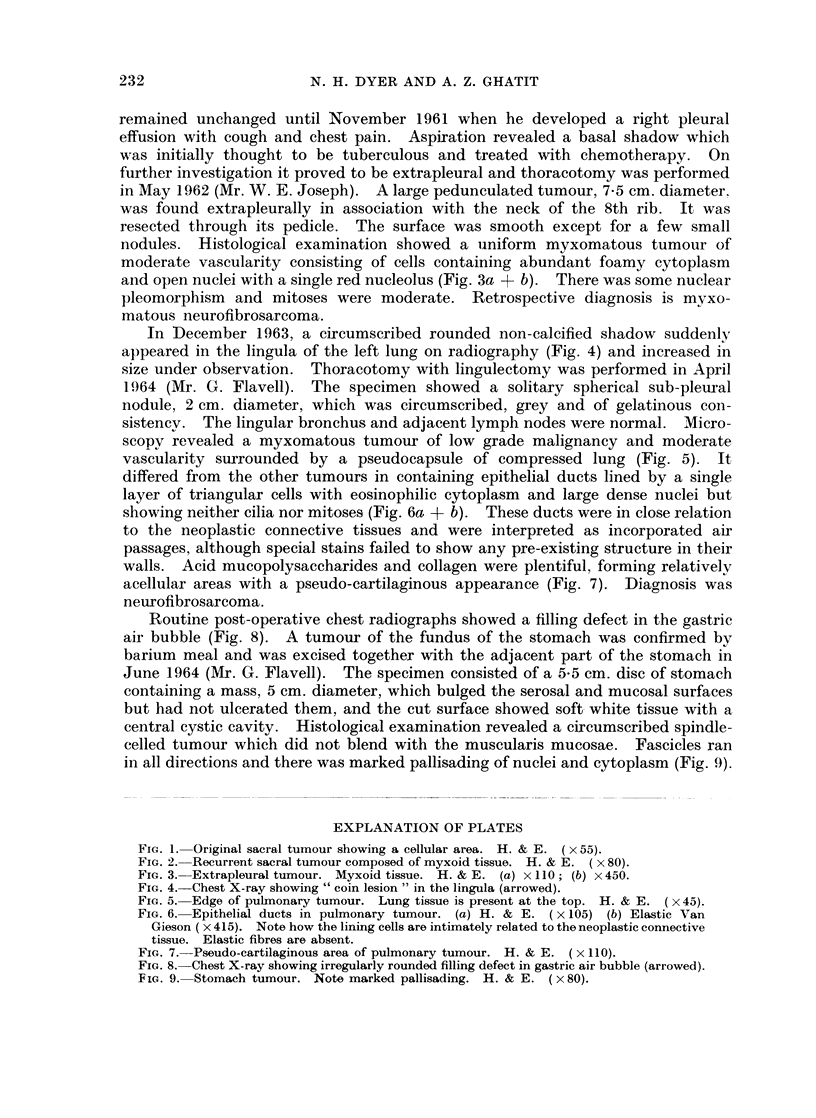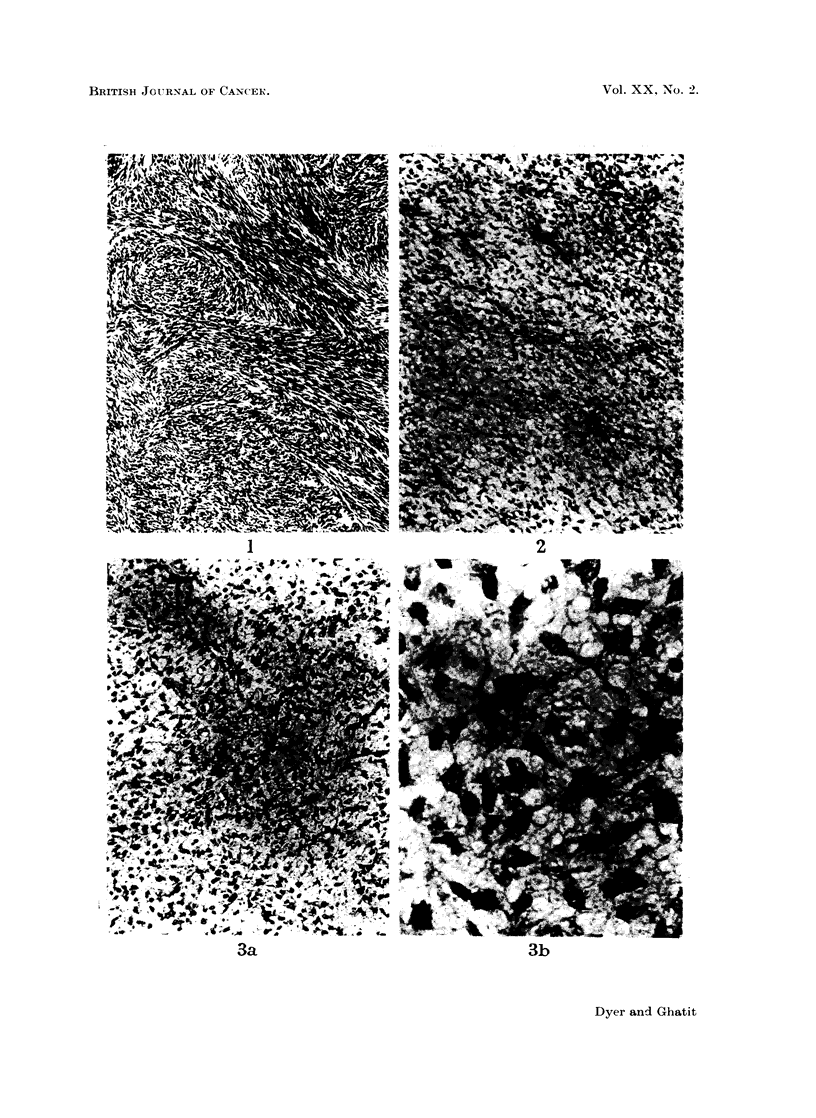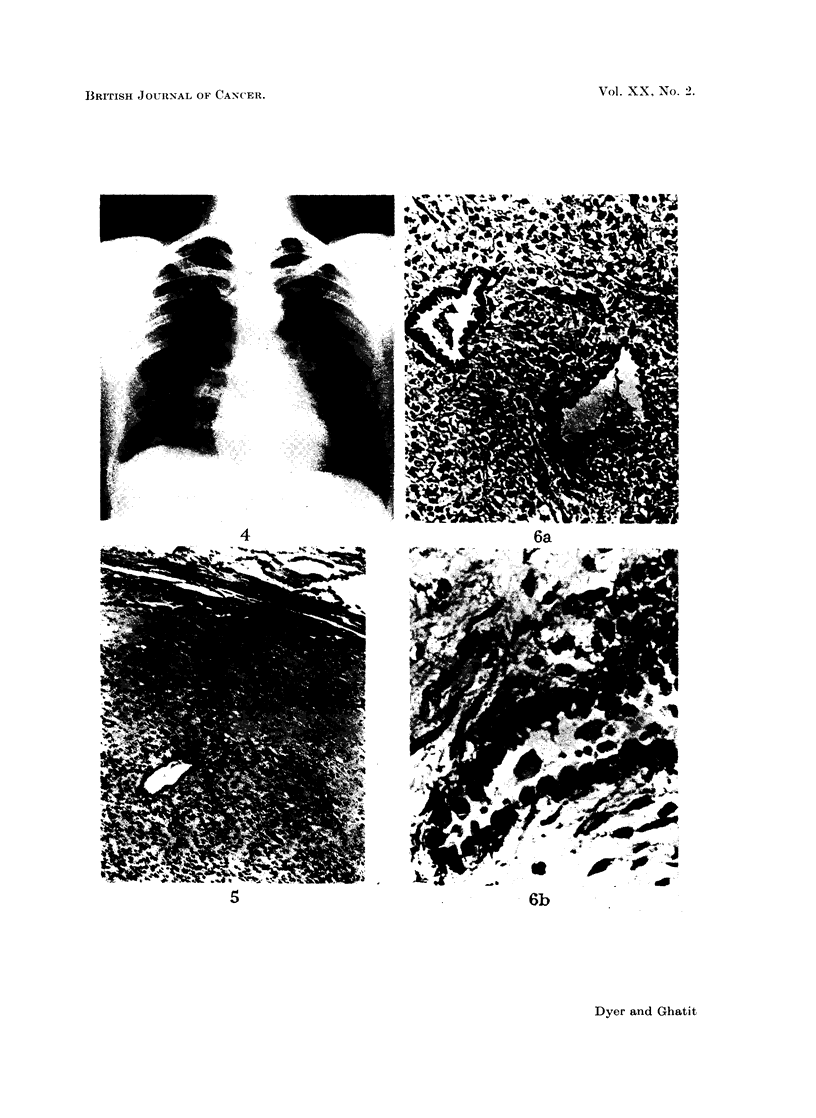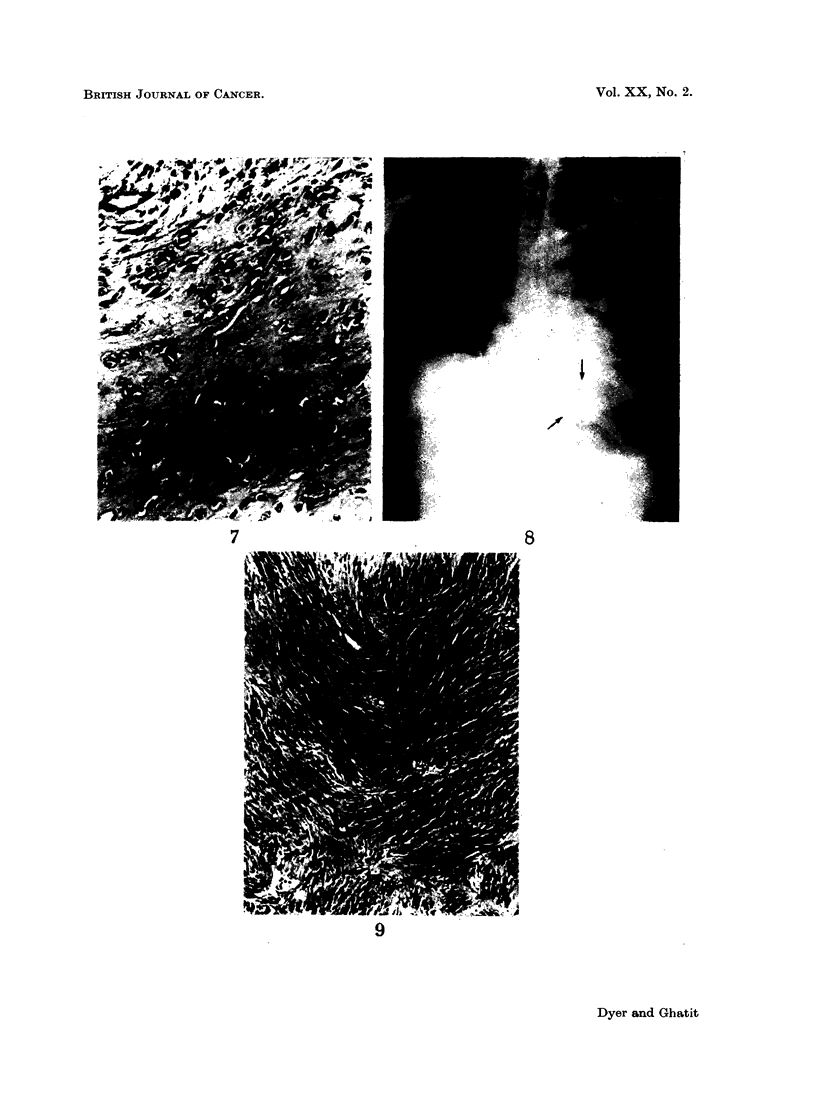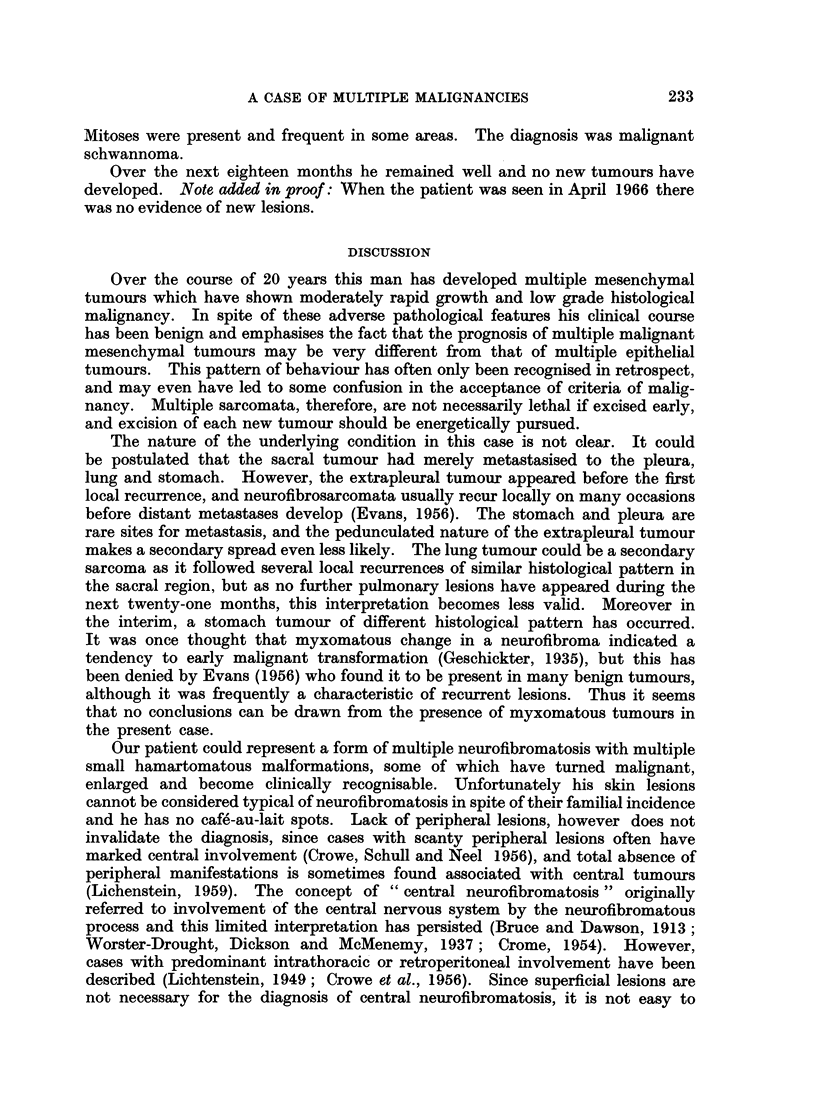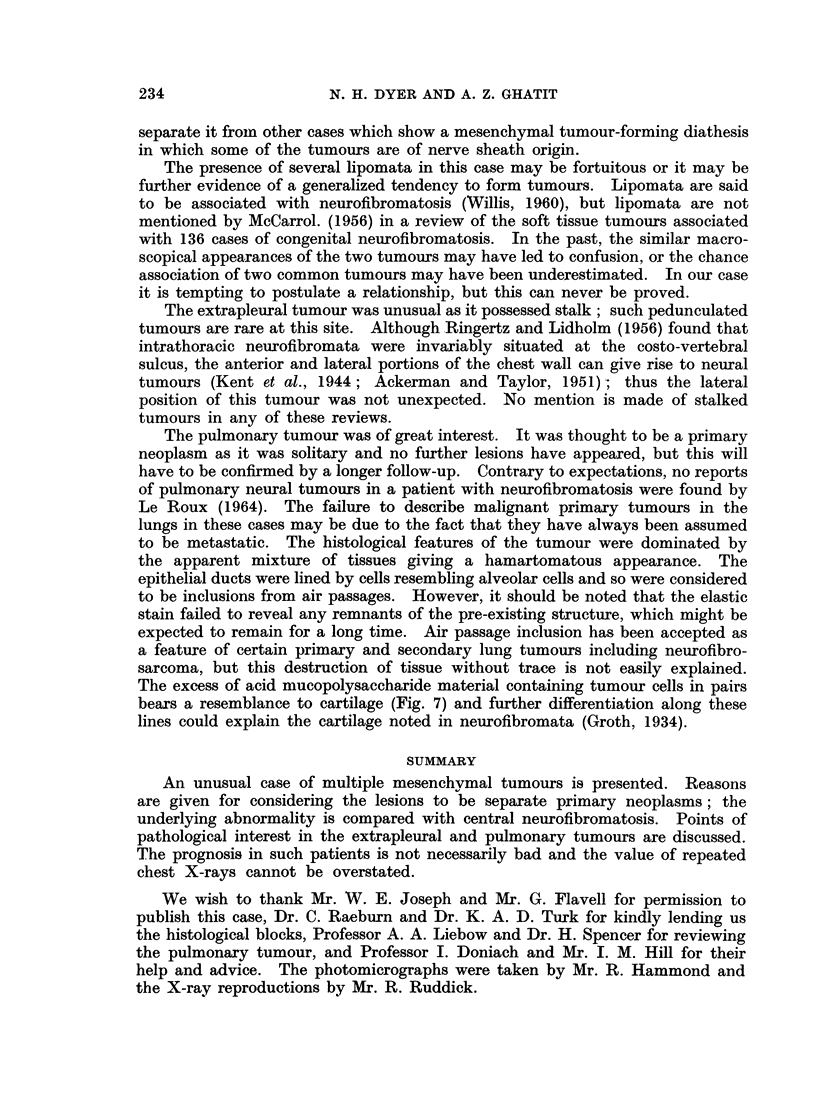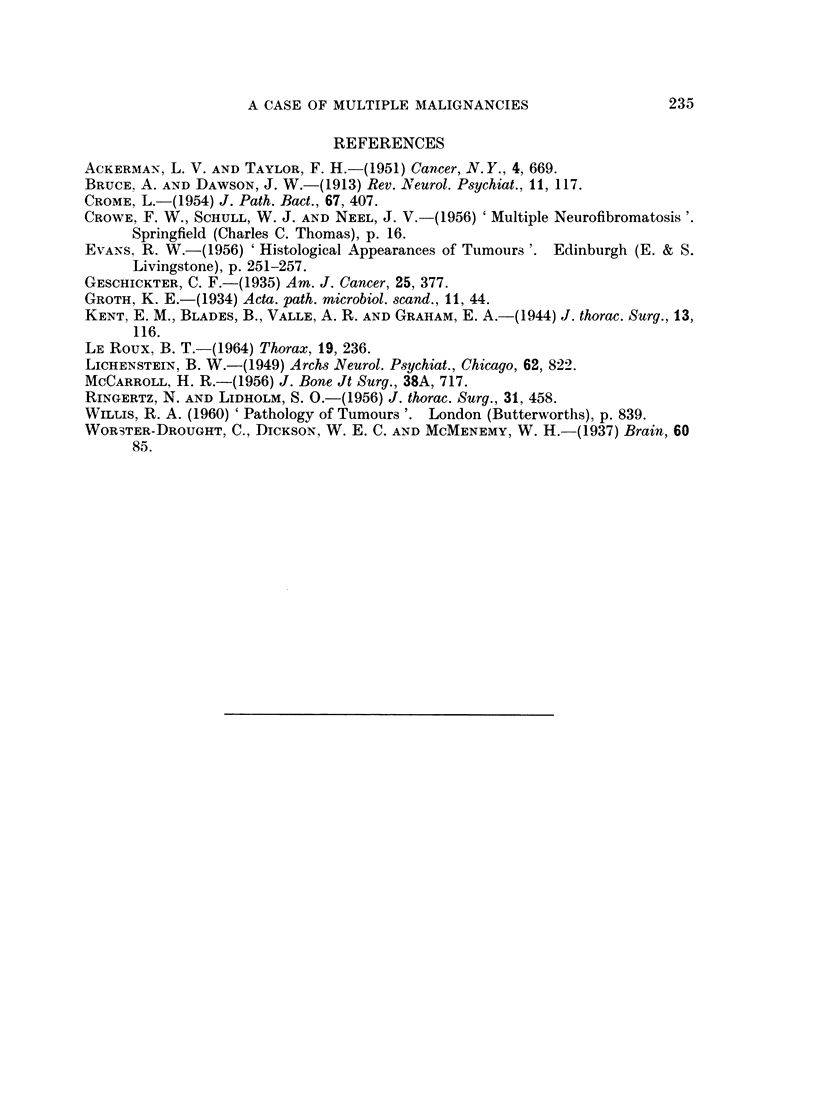# A case of multiple malignant tumours successfully treated by surgery.

**DOI:** 10.1038/bjc.1966.28

**Published:** 1966-06

**Authors:** N. H. Dyer, A. Z. Ghatit

## Abstract

**Images:**


### A CASE OF MULTIPLE MALIGNANT TUMOURS SUCCESSFULLY TREATED BY SURGERY

N. H. DYER AND A. Z. GHATIT
*From the London Hospital, London, E.*1

Received for publication March 8, 1966

SURVIVAL for a number of years with multiple malignant tumours is unusual. A case is described in which several malignant tumours, probably of nerve sheath origin, developed and certain interesting features of the clinical syndrome and histology are discussed.

### CASE REPORT

The patient, a G.P.O. Engineer, was born in 1921. He has no deformities, but a 3 cm. recurrent subcutaneous lipoma is present over the left deltoid muscle and his skin contains a moderate number of small pedunculated polypi situated over his trunk. There are no café-au-lait spots or subcutaneous neurofibromata. He stated that his father had had similar skin lesions.

In 1941 a mobile mass was excised from the left shoulder region and was said to be a lipoma. Four years later further similar lesions appeared on the right shoulder and on the dorsum of the left hand and were eventually excised in 1962, when histological examination of the former revealed a benign lipoma. At this time a further long-standing swelling was removed from the left side of the neck and found to be an epidermal cyst on microscopy.

In 1945 a small subcutaneous mass appeared over the sacrum and gradually enlarged to 8 × 8 cm. but remained circumscribed and mobile. It was excised in 1961 by Mr. W. E. Joseph. Histological examination showed a cellular tumour with some areas of increased cellularity consisting of tangled interlacing fascicles of wavy spindle cells with sharp-ended nuclei (Fig. 1). There was some alignment of nuclei, but pallisading was not prominent. Mitoses were present and moderately frequent in some areas. The background material was an acid mucopolysaccharide, which varied in amounts in different sections. Nerve fibres were not demonstrated. Occasional mast cells and foci of lymphocytes were seen. The tumour had an indefinite edge and appeared to be invasive ; it was thought to be a solitary neurofibrosarcoma.

A few months later the sacral mass recurred, rapidly attained 10 cm. diameter and was again excised in 1962 (Mr. W. E. Joseph). The tumour reappeared a third time in 1963, was again excised and a course of radiotherapy was given. There has been no further recurrence. The microscopical appearance of the recurrent tumour was of an entirely myxomatous, vascular growth showing no obvious " neural " pattern (Fig. 2). There were more mitoses than previously and acid mucopolysaccharides were more plentiful. This appearance was very different from the original tumour but similar to the extrapleural tumour (*vide infra*). The diagnosis was recurrent myxomatous neurofibrosarcoma.

Meanwhile, in 1949, old bilateral apical pulmonary tuberculosis had been discovered by miniature mass X-ray. Since then yearly chest radiography remained unchanged until November 1961 when he developed a right pleural effusion with cough and chest pain. Aspiration revealed a basal shadow which was initially thought to be tuberculous and treated with chemotherapy. On further investigation it proved to be extrapleural and thoracotomy was performed in May 1962 (Mr. W. E. Joseph). A large pedunculated tumour, 7·5 cm. diameter, was found extrapleurally in association with the neck of the 8th rib. It was resected through its pedicle. The surface was smooth except for a few small nodules. Histological examination showed a uniform myxomatous tumour of moderate vascularity consisting of cells containing abundant foamy cytoplasm and open nuclei with a single red nucleolus (Fig. 3*a* + *b*). There was some nuclear pleomorphism and mitoses were moderate. Retrospective diagnosis is myxomatous neurofibrosarcoma.

In December 1963, a circumscribed rounded non-calcified shadow suddenly appeared in the lingula of the left lung on radiography (Fig. 4) and increased in size under observation. Thoracotomy with lingulectomy was performed in April 1964 (Mr. G. Flavell). The specimen showed a solitary spherical sub-pleural nodule, 2 cm. diameter, which was circumscribed, grey and of gelatinous consistency. The lingular bronchus and adjacent lymph nodes were normal. Microscopy revealed a myxomatous tumour of low grade malignancy and moderate vascularity surrounded by a pseudocapsule of compressed lung (Fig. 5). It differed from the other tumours in containing epithelial ducts lined by a single layer of triangular cells with eosinophilic cytoplasm and large dense nuclei but showing neither cilia nor mitoses (Fig. 6*a* + *b*). These ducts were in close relation to the neoplastic connective tissues and were interpreted as incorporated air passages, although special stains failed to show any pre-existing structure in their walls. Acid mucopolysaccharides and collagen were plentiful, forming relatively acellular areas with a pseudo-cartilaginous appearance (Fig. 7). Diagnosis was neurofibrosarcoma.

Routine post-operative chest radiographs showed a filling defect in the gastric air bubble (Fig. 8). A tumour of the fundus of the stomach was confirmed by barium meal and was excised together with the adjacent part of the stomach in June 1964 (Mr. G. Flavell). The specimen consisted of a 5·5 cm. disc of stomach containing a mass, 5 cm. diameter, which bulged the serosal and mucosal surfaces but had not ulcerated them, and the cut surface showed soft white tissue with a central cystic cavity. Histological examination revealed a circumscribed spindle-celled tumour which did not blend with the muscularis mucosae. Fascicles ran in all directions and there was marked pallisading of nuclei and cytoplasm (Fig. 9).

### EXPLANATION OF PLATES

FIG. 1.—Original sacral tumour showing a cellular area. H. & E. (×55).
FIG. 2.—Recurrent sacral tumour composed of myxoid tissue. H. & E. (×80).
FIG. 3.—Extrapleural tumour. Myxoid tissue. H. & E. (*a*) ×110; (*b*) ×450.
FIG. 4.—Chest X-ray showing " coin lesion " in the lingula (arrowed).
FIG. 5.—Edge of pulmonary tumour. Lung tissue is present at the top. H. & E. (×45).
FIG. 6.—Epithelial ducts in pulmonary tumour. (*a*) H. & E. (×105) (*b*) Elastic Van Gieson (×415). Note how the lining cells are intimately related to the neoplastic connective tissue. Elastic fibres are absent.
FIG. 7.—Pseudo-cartilaginous area of pulmonary tumour. H. & E. (×110).
FIG. 8.—Chest X-ray showing irregularly rounded filling defect in gastric air bubble (arrowed).
FIG. 9.—Stomach tumour. Note marked pallisading. H. & E. (×80).

1

2

3a

3b

Dyer and Ghatit

4

6a

5

6b

Dyer and Ghatit

7

8

9

Dyer and Ghatit

Mitoses were present and frequent in some areas. The diagnosis was malignant schwannoma.

Over the next eighteen months he remained well and no new tumours have developed. *Note added in proof:* When the patient was seen in April 1966 there was no evidence of new lesions.

### DISCUSSION

Over the course of 20 years this man has developed multiple mesenchymal tumours which have shown moderately rapid growth and low grade histological malignancy. In spite of these adverse pathological features his clinical course has been benign and emphasises the fact that the prognosis of multiple malignant mesenchymal tumours may be very different from that of multiple epithelial tumours. This pattern of behaviour has often only been recognised in retrospect, and may even have led to some confusion in the acceptance of criteria of malignancy. Multiple sarcomata, therefore, are not necessarily lethal if excised early, and excision of each new tumour should be energetically pursued.

The nature of the underlying condition in this case is not clear. It could be postulated that the sacral tumour had merely metastasised to the pleura, lung and stomach. However, the extrapleural tumour appeared before the first local recurrence, and neurofibrosarcomata usually recur locally on many occasions before distant metastases develop (Evans, 1956). The stomach and pleura are rare sites for metastasis, and the pedunculated nature of the extrapleural tumour makes a secondary spread even less likely. The lung tumour could be a secondary sarcoma as it followed several local recurrences of similar histological pattern in the sacral region, but as no further pulmonary lesions have appeared during the next twenty-one months, this interpretation becomes less valid. Moreover in the interim, a stomach tumour of different histological pattern has occurred. It was once thought that myxomatous change in a neurofibroma indicated a tendency to early malignant transformation (Geschickter, 1935), but this has been denied by Evans (1956) who found it to be present in many benign tumours, although it was frequently a characteristic of recurrent lesions. Thus it seems that no conclusions can be drawn from the presence of myxomatous tumours in the present case.

Our patient could represent a form of multiple neurofibromatosis with multiple small hamartomatous malformations, some of which have turned malignant, enlarged and become clinically recognisable. Unfortunately his skin lesions cannot be considered typical of neurofibromatosis in spite of their familial incidence and he has no café-au-lait spots. Lack of peripheral lesions, however does not invalidate the diagnosis, since cases with scanty peripheral lesions often have marked central involvement (Crowe, Schull and Neel 1956), and total absence of peripheral manifestations is sometimes found associated with central tumours (Lichenstein, 1959). The concept of " central neurofibromatosis " originally referred to involvement of the central nervous system by the neurofibromatous process and this limited interpretation has persisted (Bruce and Dawson, 1913; Worster-Drought, Dickson and McMenemy, 1937; Crome, 1954). However, cases with predominant intrathoracic or retroperitoneal involvement have been described (Lichtenstein, 1949; Crowe *et al.*, 1956). Since superficial lesions are not necessary for the diagnosis of central neurofibromatosis, it is not easy to separate it from other cases which show a mesenchymal tumour-forming diathesis in which some of the tumours are of nerve sheath origin.

The presence of several lipomata in this case may be fortuitous or it may be further evidence of a generalized tendency to form tumours. Lipomata are said to be associated with neurofibromatosis (Willis, 1960), but lipomata are not mentioned by McCarrol. (1956) in a review of the soft tissue tumours associated with 136 cases of congenital neurofibromatosis. In the past, the similar macroscopical appearances of the two tumours may have led to confusion, or the chance association of two common tumours may have been underestimated. In our case it is tempting to postulate a relationship, but this can never be proved.

The extrapleural tumour was unusual as it possessed stalk ; such pedunculated tumours are rare at this site. Although Ringertz and Lidholm (1956) found that intrathoracic neurofibromata were invariably situated at the costo-vertebral sulcus, the anterior and lateral portions of the chest wall can give rise to neural tumours (Kent *et al.*, 1944 ; Ackerman and Taylor, 1951) ; thus the lateral position of this tumour was not unexpected. No mention is made of stalked tumours in any of these reviews.

The pulmonary tumour was of great interest. It was thought to be a primary neoplasm as it was solitary and no further lesions have appeared, but this will have to be confirmed by a longer follow-up. Contrary to expectations, no reports of pulmonary neural tumours in a patient with neurofibromatosis were found by Le Roux (1964). The failure to describe malignant primary tumours in the lungs in these cases may be due to the fact that they have always been assumed to be metastatic. The histological features of the tumour were dominated by the apparent mixture of tissues giving a hamartomatous appearance. The epithelial ducts were lined by cells resembling alveolar cells and so were considered to be inclusions from air passages. However, it should be noted that the elastic stain failed to reveal any remnants of the pre-existing structure, which might be expected to remain for a long time. Air passage inclusion has been accepted as a feature of certain primary and secondary lung tumours including neurofibrosarcoma, but this destruction of tissue without trace is not easily explained. The excess of acid mucopolysaccharide material containing tumour cells in pairs bears a resemblance to cartilage (Fig. 7) and further differentiation along these lines could explain the cartilage noted in neurofibromata (Groth, 1934).

### SUMMARY

An unusual case of multiple mesenchymal tumours is presented. Reasons are given for considering the lesions to be separate primary neoplasms ; the underlying abnormality is compared with central neurofibromatosis. Points of pathological interest in the extrapleural and pulmonary tumours are discussed. The prognosis in such patients is not necessarily bad and the value of repeated chest X-rays cannot be overstated.

We wish to thank Mr. W. E. Joseph and Mr. G. Flavell for permission to publish this case, Dr. C. Raeburn and Dr. K. A. D. Turk for kindly lending us the histological blocks, Professor A. A. Liebow and Dr. H. Spencer for reviewing the pulmonary tumour, and Professor I. Doniach and Mr. I. M. Hill for their help and advice. The photomicrographs were taken by Mr. R. Hammond and the X-ray reproductions by Mr. R. Ruddick.

### REFERENCES

ACKERMAN, L. V. AND TAYLOR, F. H.—(1951) *Cancer, N.Y.*, **4**, 669.
BRUCE, A. AND DAWSON, J. W.—(1913) *Rev. Neurol. Psychiat.*, **11**, 117.
CROME, L.—(1954) *J. Path. Bact.*, **67**, 407.
CROWE, F. W., SCHULL, W. J. AND NEEL, J. V.—(1956) ' Multiple Neurofibromatosis '. Springfield (Charles C. Thomas), p. 16.
EVANS, R. W.—(1956) ' Histological Appearances of Tumours '. Edinburgh (E. & S. Livingstone), p. 251–257.
GESCHICKTER, C. F.—(1935) *Am. J. Cancer*, **25**, 377.
GROTH, K. E.—(1934) *Acta. path. microbiol. scand.*, **11**, 44.
KENT, E. M., BLADES, B., VALLE, A. R. AND GRAHAM, E. A.—(1944) *J. thorac. Surg.*, **13**, 116.
LE ROUX, B. T.—(1964) *Thorax*, **19**, 236.
LICHENSTEIN, B. W.—(1949) *Archs Neurol. Psychiat., Chicago*, **62**, 822.
McCARROLL, H. R.—(1956) *J. Bone Jt Surg.*, **38**A, 717.
RINGERTZ, N. AND LIDHOLM, S. O.—(1956) *J. thorac. Surg.*, **31**, 458.
WILLIS, R. A. (1960) ' Pathology of Tumours '. London (Butterworths), p. 839.
WORSTER-DROUGHT, C., DICKSON, W. E. C. AND McMENEMY, W. H.—(1937) *Brain*, **60** 85.